# Risk factors of methicillin-resistant *Staphylococcus aureus* colonization in the nasal cavity of people living with HIV: a cross-sectional study from Dongyang hospital, Zhejiang Province

**DOI:** 10.3389/fmicb.2025.1634460

**Published:** 2025-08-13

**Authors:** Dongping Hu, Li Hong, Tinghua Ye, Bin Yang, Lixia Wang

**Affiliations:** ^1^Infectious Disease Department, Affiliated Dongyang Hospital of Wenzhou Medical University, Dongyang, China; ^2^Department of Laboratory, Affiliated Dongyang Hospital of Wenzhou Medical University, Dongyang, China; ^3^Department of Infectious Diseases, Shanghai Xuhui Central Hospital/Zhongshan-Xuhui Hospital, Fudan University, Shanghai, China; ^4^Department of Urology, Affiliated Dongyang Hospital of Wenzhou Medical University, Dongyang, China

**Keywords:** MRSA, people living with HIV, risk factors, nasal carriage, drug resistance

## Abstract

**Objective:**

To investigate the distribution characteristics of methicillin-resistant *Staphylococcus aureus* (MRSA) infection in people living with HIV (PLWH), to analyse the risk factors of MRSA colonisation in the nasopharynx of PLWH, and to provide a scientific basis for the prevention of hospital-acquired MRSA infection in PLWH.

**Methods:**

This study used a cross-sectional research design to analyse 1,100 PLWH attending the AIDS outpatient clinic of the People’s Hospital of Dongyang City, Zhejiang Province, from January 2022 to December 2024. Nasal swabs were collected with informed consent, and epidemiological information was collected via questionnaire. Standard microbiological methods were used for isolation and identification of strains, with drug susceptibility testing performed using the K-B paper diffusion method. PCR was used to detect virulence genes *pvl* and *tst.* Statistical analysis was conducted using Stata13.0 software.

**Results:**

Of the 1,100 PLWH enrolled, 275 (25%) were colonized with *S. aureus*, of which 98 (35.63%) carried MRSA and 177 (64.37%) carried MSSA. The mean age of MRSA carriers (51.32 ± 15.87 years) was significantly higher than that of MSSA carriers (44.26 ± 18.93 years) (*p* < 0.001). MRSA had a high prevalence of resistance to a wide range of antibiotics, including penicillin, oxacillin, amoxicillin and amoxicillin/clavulanic acid. The virulence gene *pvl* was detected more frequently in MSSA than in MRSA. In PLWH, respiratory infections in the previous 12 months, lack of antiretroviral therapy and heterosexual transmission were associated with a higher risk of nasal carriage of MRSA.

**Conclusion:**

The study provides valuable insights into the characteristics and distribution of MRSA and MSSA cases, as well as the factors influencing the nasal carriage of *Staphylococcus aureus*, particularly MRSA, in PLWH. These findings can guide clinical practice and infection control measures to reduce the incidence and spread of MRSA infections in high-risk populations.

## 1 Introduction

Methicillin-Resistant *Staphylococcus aureus* (MRSA) is a global public health problem that can cause multi-organ, skin and soft tissue infections and increase the risk of death in hospital settings (Chalmers and Wylam, 2020). The colonisation and infection rates of *Staphylococcus aureus* (*S. aureus*) and MRSA in people living with HIV (PLWH) are significantly higher than those in non-HIV-infected individuals (Sabbagh et al., 2019; Hsu et al., 2020), making this population particularly vulnerable to complications from these infections (Reid et al., 2017b).

The rate of nasal MRSA colonisation in PLWH differs between countries and regions, with CC30-ST36 being common in the United States and the United Kingdom, CC45 predominant in Europe, and CC5-ST5 and CC22-ST22 most common in Asian countries (Bhattacharya and Horswill, 2024). Studies of MRSA susceptibility in nasopharyngeal screening of PLWH from Taiwan and Brazil showed significant differences in drug resistance to sulfamethoxazole-trimethoprim, suggesting regional differences in MRSA strains carried by PLWH (Popovich et al., 2013).

Studies show that MRSA is predominantly nasal in PLWH. In North America, the prevalence of nasal carriage was 8.8%, with the risk of MRSA in PLWH with a history of hospitalisation in the past 12 months being 3.1 times higher than in those without (Donkor et al., 2019). Results from Atlanta showed an overall nasal and axillary carriage rate of 13% (Kotpal et al., 2016), while a Johns Hopkins University study showed a 15.4% prevalence of MRSA in PLWH, with 59.7% from the nasal cavity (Zervou et al., 2014).

The prevalence of *S. aureus* or MRSA varies regionally, possibly related to differences in strain virulence, colonisation properties, antimicrobial drug utilization, and infection control methods. Risk factors for MRSA colonization in PLWH have been reported to include CD4 cell counts, history of infections, hospitalization, antibiotic use, and demographic factors, though findings are inconsistent across studies (Peters et al., 2011; Popovich et al., 2013; Farley et al., 2015).

Currently, the prevalence of MRSA in nasopharyngeal colonisation of PLWH in Zhejiang Province has not been reported, and there is a lack of drug susceptibility data for this regional population. Therefore, it is necessary to investigate the prevalence of MRSA in nasopharyngeal colonisation of PLWH in this region, analyze its drug resistance characteristics, and identify risk factors associated with MRSA colonization in this specific population.

The primary objective of this study is to determine the prevalence of nasal MRSA colonization among PLWH in Zhejiang Province and to identify associated risk factors. The secondary objectives are to characterize the antimicrobial resistance patterns and virulence factors of MRSA isolates from this population.

## 2 Methods

### 2.1 Ethics statement

The study was approved by the Ethics and Programme Review Committee of the People’s Hospital of Dongyang City, Zhejiang Province, under the ethical approval number “DYRM2022-135.”

### 2.2 Research design

In this study, a cross-sectional research design was used to collect nasal swab specimens from PLWH and a questionnaire was administered using a combination of face-to-face and self-administered methods. The study was conducted from January 2022 to December 2024.

### 2.3 Inclusion and exclusion criteria

#### 2.3.1 Inclusion criteria

(1) Patients aged ≥18 years who are HIV-positive in the initial screening and confirmatory test who voluntarily submit to the investigation; (2) *Staphylococcus aureus* detected as MRSA by oxacillin disk assay and mecA gene PCR.

#### 2.3.2 Exclusion criteria

(1) Patients with MRSA infection detected by admission screening; (2) Patients not screened for MRSA on admission or within 48 h; (3) Patients with MRSA infection detected within 48 h of admission; (4) Patients who died, were discharged without healing, or discharged voluntarily; (5) Patients with hospital stay less than 48 h; (6) Patients without weekly MRSA screening; (7) Patients transferred after treatment at other hospitals; (8) Patients with incomplete clinical information.

### 2.4 Sample collection and processing

A total of 1,100 PLWH were enrolled in the study, and one nasal swab specimen was collected from each participant. Nasal swabs were collected by trained personnel following standard protocols. Briefly, sterile cotton swabs moistened with sterile saline were used to sample the bilateral nasal vestibules of subjects. Samples were placed in sterile tubes containing 7.5% NaCl broth and transported to the laboratory within 6 h for processing.

### 2.5 Laboratory methods

*Staphylococcus aureus* identification was performed using mannitol salt agar (MSA, BD Difco™, USA) as a selective medium to differentiate *S. aureus* from coagulase-negative staphylococci such as *S. epidermidis*. *S. aureus* identification and drug susceptibility testing were performed according to standard clinical microbiological procedures. The Kirby-Bauer (K-B) disk diffusion method was used for antimicrobial susceptibility testing according to Clinical and Laboratory Standards Institute (CLSI) 2023 guidelines (Clinical and Laboratory Standards Institute, 2023). MRSA identification was performed using both phenotypic and genotypic methods. For phenotypic identification, cefoxitin (30 μg) disk diffusion was used, with an inhibition zone diameter ≤21 mm indicating MRSA. For genotypic confirmation, PCR detection of the *mecA* gene was performed using the following primers: *mecA*-F: 5’-GTAGAAATGACTGAACGTCCGATAA-3′ and *mecA*-R: 5’-CCAATTCCACATTGTTTCGGTCTAA-3′, with an expected product size of 310 bp.

### 2.6 Statistical analysis

Categorical data were expressed as frequencies and percentages; normally distributed data were expressed as mean and standard deviation; and non-normally distributed data were expressed as median and interquartile range. For the comparison of characteristics between MRSA and MSSA carriers, chi-square tests were used for categorical variables and t-tests or Wilcoxon rank-sum tests were used for continuous variables, as appropriate. All percentages were calculated based on the actual number of subjects in each group (MRSA *n* = 98, MSSA *n* = 177).

Unconditional logistic regression models were used to investigate risk factors for MRSA colonization among *S. aureus* carriers. The odds ratio (OR) and 95% confidence interval (95%CI) were calculated for each factor, with factors showing *p* < 0.20 in univariate analysis included in the multivariate model.

## 3 Results

### 3.1 Demographic characteristics of MRSA and MSSA patients

Of the 1,100 PLWH enrolled, 275 (25%) were colonized with *S. aureus*, of which 98 (35.63%) carried MRSA and 177 (64.37%) carried MSSA (see Table 1).

**Table 1:** Gender and age distribution in MRSA and MSSA Groups [*n* (%)].

Characteristic	MRSA (*n* = 98)	MSSA (*n* = 177)	*t*/*χ*^2^	*p*
Gender			1.023	0.312
Male	54 (55.10)	88 (49.71)		
Female	44 (44.90)	89 (50.29)		
Age (mean ± SD)	**51.32 ± 15.87**	**44.26 ± 18.93**	**−3.156**	**0.002**
Age groups				
18–29	**11 (11.22)**	**62 (35.03)**	**17.892**	**<0.001**
30–39	**12 (12.24)**	**28 (15.82)**	0.687	0.407
40–49	**16 (16.33)**	**29 (16.38)**	0.001	0.971
50–59	**28 (28.57)**	**31 (17.51)**	**4.567**	**0.033**
60–69	**20 (20.41)**	**17 (9.60)**	**6.543**	**0.011**
≥70	**11 (11.22)**	**10 (5.65)**	**2.901**	0.089

Values in parentheses represent percentages. MRSA, methicillin-resistant *Staphylococcus aureus*; MSSA, methicillin-sensitive *Staphylococcus aureus*.

The bold values indicate statistically significant results (*p* < 0.05).

The gender distribution between MRSA and MSSA groups showed no significant difference (*p* = 0.312). However, the mean age of MRSA carriers (51.32 ± 15.87 years) was significantly higher than that of MSSA carriers (44.26 ± 18.93 years) (*p* = 0.002). Age group analysis showed that MSSA was significantly more common in younger age groups (18–29 years), while MRSA was more prevalent in older age groups (50–59 and 60–69 years) (see Table 2).

**Table 2:** Antibiotic resistance rates comparison between MRSA and MSSA.

Antibiotic	MRSA (*n* = 98)	MSSA (*n* = 177)	*χ* ^2^	*p*
Penicillin	98 (100.00)	153 (86.44)	14.444	<0.001
Oxacillin	98 (100.00)	0 (0.00)	275.000	<0.001
Amoxicillin	96 (98.00)	148 (83.62)	13.156	<0.001
Amoxicillin/Clavulanic Acid	82 (83.67)	0 (0.00)	196.849	<0.001
Cefazolin	80 (81.63)	0 (0.00)	189.872	<0.001
Imipenem	78 (80.00)	0 (0.00)	183.273	<0.001
Vancomycin	0 (0.00)	0 (0.00)	–	–
Gentamicin	58 (59.18)	13 (7.34)	89.544	<0.001
Erythromycin	85 (86.73)	61 (34.46)	68.756	<0.001
Rifampicin	10 (10.20)	2 (1.13)	12.067	<0.001
Tetracycline	68 (69.39)	48 (27.12)	46.444	<0.001
Ciprofloxacin	64 (65.31)	27 (15.25)	71.644	<0.001
Levofloxacin	76 (77.55)	126 (71.19)	1.256	0.262
Clindamycin	64 (65.31)	41 (23.16)	47.256	<0.001
Chloramphenicol	24 (24.49)	14 (7.91)	14.733	<0.001
Quinupristin/Dalfopristin	6 (6.12)	2 (1.13)	5.444	0.020
Linezolid	0 (0.00)	0 (0.00)	–	–
Trimethoprim/Sulfamethoxazole	24 (24.49)	0 (0.00)	48.289	<0.001

Values in parentheses represent percentages. MRSA, methicillin-resistant *Staphylococcus aureus*; MSSA, methicillin-sensitive *Staphylococcus aureus*.

### 3.2 Distribution of MRSA resistance to antibiotics

MRSA isolates showed 100% resistance to penicillin and oxacillin, with high resistance rates to amoxicillin (98%), amoxicillin/clavulanic acid (83.67%), cefazolin (81.63%), and imipenem (80%). MRSA exhibited significantly higher resistance rates than MSSA to most antibiotics tested (*p* < 0.001), with the exception of levofloxacin (*p* = 0.262). Notably, no resistance to vancomycin and linezolid was detected in either MRSA or MSSA isolates (see Table 3).

**Table 3:** *Staphylococcus aureus* virulence genes analysis.

Gene	MRSA (*n* = 98)	MSSA (*n* = 177)	*χ* ^2^	*p*
pvl**+**	8 (8.16)	45 (25.42)	12.627	<0.001
tst**+**	12 (12.24)	18 (10.17)	0.281	0.596

Values in parentheses represent percentages. MRSA, methicillin-resistant *Staphylococcus aureus*; MSSA, methicillin-sensitive *Staphylococcus aureus*. *pvl*, Panton-Valentine Leukocidin gene; *tst*, toxic shock syndrome toxin-1 gene.

### 3.3 Detection of virulence genes

The *pvl* gene was detected at a significantly lower rate in MRSA isolates (8.16%) compared to MSSA isolates (25.42%) (*p* < 0.001). In contrast, the prevalence of the *tst* gene showed no significant difference between MRSA (12.24%) and MSSA (10.17%) isolates (*p* = 0.596) (see Table 4).

**Table 4:** Univariate analysis of factors associated with MRSA nasal colonization among *S. aureus* carriers.

Factor	MRSA (*n* = 98)	MSSA (*n* = 177)	*χ* ^2^	*p*-value
	*n* (%)	*n* (%)		
Marital status			1.728	0.632
Unmarried	**18 (18.37)**	33 (18.64)		
Married	**30 (30.61)**	52 (29.38)		
Divorced or Widowed	**50 (51.02)**	92 (51.98)		
Education level			2.010	0.571
Primary or Below	**20 (20.41)**	35 (19.77)		
Junior High	**22 (22.45)**	40 (22.60)		
High School/Vocational	**28 (28.57)**	47 (26.55)		
College or Above	**28 (28.57)**	55 (31.07)		
Sharing living items			0.060	0.807
Yes	**20 (20.41)**	38 (21.47)		
No	**78 (79.59)**	139 (78.53)		
Hospitalization in past year			1.200	0.549
Yes	**45 (45.92)**	73 (41.24)		
No	**53 (54.08)**	104 (58.76)		
Respiratory infection in past 12 months			**6.850**	**0.009**
Yes	**42 (42.86)**	48 (27.12)		
No	**56 (57.14)**	129 (72.88)		
Antibiotic use in past 12 months			0.450	0.503
Yes	**45 (45.92)**	88 (49.72)		
No	**53 (54.08)**	89 (50.28)		
Skin, wound, or joint disease in past 12 months			0.250	0.618
Yes	**32 (32.65)**	52 (29.38)		
No	**66 (67.35)**	125 (70.62)		
Other infectious disease history			0.160	0.690
Yes	**28 (28.57)**	46 (25.99)		
No	**70 (71.43)**	131 (74.01)		
Opportunistic infection history (healed)			0.010	0.920
Yes	**45 (45.92)**	80 (45.20)		
No	**53 (54.08)**	97 (54.80)		
Surgical history in past 3 years			0.120	0.730
Yes	**48 (48.98)**	82 (46.33)		
No	**50 (51.02)**	95 (53.67)		
Underlying illness history			0.001	0.971
Yes	**50 (51.02)**	91 (51.41)		
No	**48 (48.98)**	86 (48.59)		
Immune disease history			0.300	0.584
Yes	**60 (61.22)**	102 (57.63)		
No	**38 (38.78)**	75 (42.37)		
Smoking in past 6 months			1.300	0.520
Yes	**40 (40.82)**	63 (35.59)		
No	**58 (59.18)**	114 (64.41)		
Alcoholism history			0.040	0.840
Yes	**42 (42.86)**	74 (41.81)		
No	**56 (57.14)**	103 (58.19)		
Number of household members			1.010	0.909
1	**20 (20.41)**	38 (21.47)		
2	**20 (20.41)**	33 (18.64)		
3	**20 (20.41)**	37 (20.90)		
4	**20 (20.41)**	35 (19.77)		
5+	**18 (18.37)**	34 (19.21)		
Drug use history			0.300	0.584
Yes	**54 (55.10)**	92 (51.98)		
No	**44 (44.90)**	85 (48.02)		
Mode of HIV infection			**8.010**	**0.046**
Heterosexual	**40 (40.82)**	48 (27.12)		
Homosexual	**25 (25.51)**	65 (36.72)		
Sex + Injection Drug Use	**33 (33.67)**	64 (36.16)		

The bold values indicate statistically significant results (*p* < 0.05).

### 3.4 Risk factors for MRSA colonization in PLWH

In the univariate analysis comparing characteristics between MRSA carriers (*n* = 98) and MSSA carriers (*n* = 177), respiratory infection in the past 12 months (42.86% vs. 27.12%, *p* = 0.009) and mode of HIV infection (*p* = 0.046) showed statistically significant associations with MRSA colonization. Specifically, MRSA carriers had a higher proportion of respiratory infections and were more likely to have acquired HIV through heterosexual transmission (40.82% vs. 27.12%).

### 3.5 Multifactorial analysis of risk factors

Based on the corrected univariate analysis, variables with *p* < 0.20 were included in the multifactorial logistic regression model to identify independent risk factors for MRSA colonization among *S. aureus* carriers (see Table 5).

**Table 5:** Multifactorial analysis of risk factors for nasal carriage of MRSA in PLWH.

Risk factor	*β*	SE	Wald *χ*^2^	*p*-value	OR	95% CI
Ethnicity						
Minority				1.000		
Han	0.971	0.751	1.29	0.196	2.642	0.607–11.507
Marital status						
Unmarried				1.000		
Married	−0.157	0.343	−0.46	0.646	0.855	0.437–1.673
Divorced or bereaved	0.004	0.530	0.01	0.994	1.004	0.355–2.836
Education level						
Primary school or below				1.000		
Junior high school	0.237	0.511	0.46	0.643	1.268	0.465–3.453
Senior high school/vocational school	−0.389	0.561	−0.69	0.488	0.678	0.226–2.035
College or above	0.060	0.541	0.11	0.911	1.062	0.368–3.065
Respiratory infection in the last 12 months						
No				1.000		
Yes	0.715	0.276	2.59	**0.010**	**2.044**	**1.191–3.509**
Smoking in the last 6 months						
No				1.000		
Yes	−0.322	0.260	−1.24	0.215	0.724	0.435–1.206
Currently using antiretroviral therapy						
Yes				1.000		
No	0.758	0.332	2.28	**0.022**	**2.133**	**1.113–4.089**
Infection route						
Homosexual transmission				1.000		
Heterosexual transmission	1.228	0.613	2.00	**0.045**	**3.413**	**1.026–11.353**
Sexual transmission + intravenous DRUG use	0.235	0.308	0.76	0.445	1.265	0.691–2.315
Other	0.450	0.543	0.83	0.404	1.572	0.543–4.553

Statistically significant values (*p* < 0.05) are highlighted in bold. MRSA, methicillin-resistant *Staphylococcus aureus*; OR, odds ratio; CI, confidence interval; SE, standard error.

Multifactorial logistic regression analysis identified three independent risk factors for MRSA nasal carriage in PLWH: respiratory infection in the previous 12 months (OR = 2.044, 95% CI: 1.191–3.509, *p* = 0.010), not receiving antiretroviral therapy (OR = 2.133, 95% CI: 1.113–4.089, *p* = 0.022), and heterosexual transmission of HIV (OR = 3.413, 95% CI: 1.026–11.353, *p* = 0.045).

## 4 Discussion

*Staphylococcus aureus* is an important pathogen causing infections in PLWH and AIDS patients. PLWH are a group with a high prevalence of *S. aureus* and MRSA carriage and infection (Vyas et al., 2011). Our study found a 25% nasal carrier rate of *S. aureus* among PLWH, with 35.63% of these being MRSA carriers, which is comparable to some international studies but with a notably higher MRSA proportion.

Previous studies have shown varying carriage rates of *S. aureus* and MRSA among different geographical areas and populations. In Texas, USA, the prevalence of *S. aureus* among PLWH was 29.7%, with MRSA accounting for 3% (Hidron et al., 2011). In Northern Ethiopia, *S. aureus* was isolated from 32.5% of respondents and MRSA from 2.4% (Gebremedhn et al., 2016). African regions generally show higher rates, with a study in Botswana reporting 55% prevalence of *S. aureus* in PLWH (Reid et al., 2017a, b). Studies from the United States show *S. aureus* detection rates of 36–39% among PLWH, with MRSA rates of 3.2–6% (Sayana et al., 2010; Lee et al., 2013). In Brazil, nasal *S. aureus* carriage was reported at 34.2% with MRSA colonization at 10.4% (Vieira et al., 2016), while a study in Iran found MRSA nasal carriage at 12.8% (Hassanzadeh et al., 2015). A study from Taiwan reported that the nasal carriage rate of *S. aureus* in PLWH was 21.5%, with an MRSA carriage rate of 3.4% (Hsu et al., 2020).

Our study revealed several important findings. First, the age distribution differed significantly between MRSA and MSSA carriers, with MRSA more common in older patients. This age-related difference may reflect different exposure risks or immune status changes with aging in PLWH. Second, MRSA isolates showed high resistance rates to multiple antibiotics, particularly beta-lactams, aminoglycosides, macrolides, and fluoroquinolones, limiting treatment options. However, all isolates remained susceptible to vancomycin and linezolid, which is encouraging for treatment of severe infections.

The higher prevalence of the *pvl* gene in MSSA compared to MRSA isolates is noteworthy, as this virulence factor is associated with increased pathogenicity, particularly in skin and soft tissue infections (Sullivan et al., 2016). This finding suggests that MSSA infections might potentially cause severe clinical manifestations despite their generally better antibiotic susceptibility profile.

Our multifactorial analysis identified three independent risk factors for MRSA nasal carriage: recent respiratory infections, lack of antiretroviral therapy, and heterosexual transmission route. Respiratory infections may provide opportunities for MRSA acquisition during healthcare encounters or may reflect compromised respiratory immunity. The association with lack of antiretroviral therapy suggests that improved immune function through effective HIV treatment may protect against MRSA colonization. The association with heterosexual transmission differs from some studies that found higher risks with homosexual transmission or injection drug use (Leung et al., 2015; Leung et al., 2017; Wu et al., 2017), and may reflect regional differences in HIV transmission patterns and associated behaviors.

## 5 Limitations

There are some limitations to this study. First, the data were derived from PLWH at a specific time and place, so the representativeness of the sample may be limited. The results may not be fully generalisable to other populations or wider geographical areas. Second, the study only reflects the factors associated with nasal carriage of *S. aureus* and its drug resistance in PLWH, but does not delve into the mechanisms of interaction between these factors. Third, the age distribution in our study population may not fully represent all PLWH in the region, as older patients may be overrepresented due to higher healthcare utilization.

## 6 Conclusion

In conclusion, our study provides important regional data on MRSA nasal colonization in PLWH in Zhejiang Province, China. The identified risk factors and antibiotic resistance patterns can guide targeted screening, infection control measures, and empirical antibiotic therapy for this vulnerable population. Future studies should explore the molecular epidemiology of these isolates and investigate intervention strategies to reduce MRSA colonization and subsequent infection risks in PLWH.

## Data Availability

The original contributions presented in the study are included in the article/Supplementary material, further inquiries can be directed to the corresponding author.
