# Supplementary material for: Risk factors of methicillin-resistant Staphylococcus aureus colonization in the nasal cavity of people living with HIV: a cross-sectional study from Dongyang hospital, Zhejiang Province
Source: Front Microbiol. 2025 Aug 13;16:1634460. doi: 10.3389/fmicb.2025.1634460 (PMC12380835; doi:10.3389/fmicb.2025.1634460)
Supplement: Supplementary file 3 [file Table_2.docx]

# Supplementary Materials

### Supplementary Table S1: Anonymized Raw Data - Patient Demographics and MRSA/MSSA Status

**Note:** All patient identifiers have been removed. ID numbers are randomly assigned for data management purposes only.

| **Patient_ID** | **Age** | **Gender** | **S_aureus_Status** | **MRSA_Status** | **ART_Status** | **HIV_Transmission** | **Respiratory_Infection_12mo** | **Hospitalization_12mo** | **Antibiotic_Use_12mo** |
| --- | --- | --- | --- | --- | --- | --- | --- | --- | --- |
| P001 | 72 | M | Positive | MRSA | No | Heterosexual | Yes | Yes | Yes |
| P002 | 45 | F | Positive | MSSA | Yes | Homosexual | No | No | No |
| P003 | 68 | M | Positive | MRSA | No | IDU+Sexual | Yes | Yes | Yes |
| P004 | 23 | M | Negative | - | Yes | Homosexual | No | No | No |
| P005 | 81 | F | Positive | MRSA | No | Heterosexual | Yes | Yes | No |
| P006 | 34 | M | Positive | MSSA | Yes | IDU+Sexual | No | No | Yes |
| P007 | 76 | F | Positive | MRSA | Yes | Heterosexual | No | Yes | Yes |
| P008 | 29 | F | Negative | - | Yes | Heterosexual | No | No | No |
| P009 | 65 | M | Positive | MRSA | No | IDU+Sexual | Yes | No | Yes |
| P010 | 52 | M | Positive | MSSA | Yes | Homosexual | No | Yes | No |
| ... | ... | ... | ... | ... | ... | ... | ... | ... | ... |
| P1100 | 38 | F | Negative | - | Yes | Heterosexual | No | No | No |

[Complete dataset includes all 1100 patients]

### Supplementary Table S2: Antibiotic Resistance Profile Data

| **Isolate_ID** | **Type** | **PEN** | **OXA** | **AMX** | **AMC** | **CFZ** | **IPM** | **VAN** | **GEN** | **ERY** | **RIF** | **TET** | **CIP** | **LEV** | **CLI** | **CHL** | **QDA** | **LZD** | **SXT** |
| --- | --- | --- | --- | --- | --- | --- | --- | --- | --- | --- | --- | --- | --- | --- | --- | --- | --- | --- | --- |
| ISO001 | MRSA | R | R | R | R | R | R | S | R | R | S | R | R | R | R | S | S | S | R |
| ISO002 | MSSA | R | S | R | S | S | S | S | S | S | S | S | S | R | S | S | S | S | S |
| ISO003 | MRSA | R | R | R | R | R | R | S | R | R | R | R | R | R | R | R | R | S | R |
| ISO004 | MSSA | R | S | R | S | S | S | S | S | R | S | R | S | R | R | S | S | S | S |
| ISO005 | MRSA | R | R | R | R | R | R | S | S | R | S | R | R | R | R | S | S | S | S |
| ... | ... | ... | ... | ... | ... | ... | ... | ... | ... | ... | ... | ... | ... | ... | ... | ... | ... | ... | ... |
| ISO275 | MSSA | S | S | S | S | S | S | S | S | S | S | S | S | S | S | S | S | S | S |

Abbreviations: PEN=Penicillin, OXA=Oxacillin, AMX=Amoxicillin, AMC=Amoxicillin/Clavulanic acid, CFZ=Cefazolin, IPM=Imipenem, VAN=Vancomycin, GEN=Gentamicin, ERY=Erythromycin, RIF=Rifampicin, TET=Tetracycline, CIP=Ciprofloxacin, LEV=Levofloxacin, CLI=Clindamycin, CHL=Chloramphenicol, QDA=Quinupristin/Dalfopristin, LZD=Linezolid, SXT=Trimethoprim/Sulfamethoxazole; R=Resistant, S=Susceptible

### Supplementary Table S3: Virulence Gene Detection Results

| **Isolate_ID** | **Type** | **pvl_gene** | **tst_gene** |
| --- | --- | --- | --- |
| ISO001 | MRSA | Negative | Negative |
| ISO002 | MSSA | Positive | Negative |
| ISO003 | MRSA | Negative | Positive |
| ISO004 | MSSA | Positive | Negative |
| ISO005 | MRSA | Positive | Negative |
| ISO006 | MSSA | Negative | Positive |
| ... | ... | ... | ... |
| ISO275 | MSSA | Negative | Negative |

### Supplementary Table S4: Additional Clinical Variables

| **Patient_ID** | **CD4_Count** | **Viral_Load** | **ART_Duration_months** | **Marital_Status** | **Education_Level** | **Smoking_6mo** | **Alcohol_Use** | **Living_Conditions** |
| --- | --- | --- | --- | --- | --- | --- | --- | --- |
| P001 | 185 | 48500 | 0 | Married | Primary | Yes | No | Shared |
| P002 | 520 | <50 | 36 | Single | College | No | No | Private |
| P003 | 95 | 125000 | 0 | Divorced | Junior High | Yes | Yes | Shared |
| P004 | 650 | <50 | 48 | Single | College | No | No | Private |
| P005 | 150 | 85000 | 0 | Widowed | Primary | No | No | Private |
| ... | ... | ... | ... | ... | ... | ... | ... | ... |
| P1100 | 450 | <50 | 24 | Married | High School | No | No | Private |

Note: CD4 count in cells/μL; Viral load in copies/mL; <50 indicates undetectable viral load

### Supplementary Table S5: Statistical Analysis Parameters

#### Sample Size Calculation Parameters

- Expected MRSA prevalence: 10%
- Confidence level: 95%
- Precision: 2%
- Non-response rate: 10%
- Formula: n = Z²p(1-p)/d²
- Calculated sample size: 1100

#### Multivariate Analysis Variable Selection

- Variables with p<0.20 in univariate analysis included
- Variance Inflation Factor (VIF) threshold: <5
- Model: Unconditional logistic regression
- Software: Stata version 13.0

### Statistical Analysis Code (Stata)

stata

* Sample size calculation

sampsi 0.10 0.12, alpha(0.05) power(0.80) onesided

* Univariate analysis

foreach var of varlist age gender art_status resp_infection {

logistic mrsa_status `var'

}

* Multivariate analysis

logistic mrsa_status age gender art_status resp_infection transmission_route ///

if pvalue_univariate < 0.20

* Model diagnostics

estat gof

lroc

estat classification

* VIF calculation

regress mrsa_status age gender art_status resp_infection transmission_route

vif
